# International health policy survey in 11 countries: assessment of non-response bias in the Norwegian sample

**DOI:** 10.1186/1472-6963-10-38

**Published:** 2010-02-10

**Authors:** Oyvind A Bjertnaes, Hilde H Iversen, Geir Bukholm

**Affiliations:** 1 Department for Quality Measurement and Patient Safety, Norwegian Knowledge Centre for the Health Services, Oslo, Norway; 2 Institute of Health Management and Health Economics, University of Oslo, Oslo, Norway

## Abstract

**Background:**

International health policy surveys are used to compare and evaluate health system performance, but little is known about the effects of non-response. The objective of this study was to assess the effects of non-response in the Norwegian part of the Commonwealth Fund international health policy survey in 2009.

**Methods:**

As part of an international health policy survey in 2009 a cross-sectional survey was conducted in Norway among a representative sample of Norwegian general practitioners. 1 400 randomly selected GPs were sent a postal questionnaire including questions about the Norwegian health care system, the quality of the GPs' own practice and the cooperation with specialist health care. The survey included three postal reminders and a telephone follow-up of postal non-respondents. The main outcome measures were increase in response rate for each reminder, the effects of demographic and practice variables on response, the effects of non-response on survey estimates, and the cost-effectiveness of each reminder.

**Results:**

After three postal reminders and one telephone follow-up, the response rate was 59.1%. Statistically significant differences between respondents and non-respondents were found for three variables; group vs. solo practice (p = 0.01), being a specialist or not (p < 0.001) and municipality centrality (least central vs. most central, p = 0.03). However, demographic and practice variables had little association with five outcome variables and the overall survey estimates changed little with additional reminders. In addition, the cost-effectiveness of the final reminders was poor.

**Conclusions:**

The response rate in the Norwegian survey was satisfactory, and the effect of non-response was small indicating adequate representativeness. The cost-effectiveness of the final reminders was poor. The Norwegian findings strengthen the international project, but restrictions in generalizability warrant further study in other countries.

## Background

Physicians and other healthcare professionals are frequently asked to participate in postal surveys, but challenges with response rates are common for such surveys [1]. In an analysis of 178 studies published in medical journals in 1991, it was found that the average response rate in physician surveys was 54%, significantly lower than the average response rate in surveys of other respondent groups [2]. Another study of a random sample of studies from 1985 to 1995 found the average response rate in physician surveys to be 61%, and 52% in large surveys with more than 1,000 observations [3]. Low response rates threaten scientific validity because it challenges the demand for representative findings [4-6]. Therefore, various methods to increase response rates have been suggested including the total design approach [7-9].

Studies reporting the effectiveness of initiatives to increase response rates are important for survey planning, but the generally low response rates in postal surveys of healthcare professionals implies that even a substantial increase in response rates is no guarantee of representative data. Therefore, systematic assessment of the effects of non-response should be conducted. There are two approaches to assess non-response bias. Securing relevant variables in the sampling frame and analyzing differences between respondents and non-respondents for these variables is one approach, another is attempting to interview non-respondents [3]. In spite of problems with response rates in postal surveys of healthcare professionals these approaches are seldom applied [3,10]. Consequently, a large part of the scientific literature using postal surveys is unable to document generalizability, a core part of scientific validity.

Norway participated in the Commonwealth Fund's international health policy survey for the first time in 2009. Eleven countries participated in the survey: Australia, Canada, France, Germany, Italy, New Zealand, Norway, Sweden, the Netherlands, United Kingdom and the United States. The surveys were conducted among primary care physicians in each country. In the Norwegian survey a random sample of Norwegian GPs was sent a postal questionnaire. Postal surveys of GPs have the same challenges with response rate as other healthcare groups [11-14]. Therefore, the data collection methods were based on recent survey research among GPs in Norway and included both multiple postal reminders and a telephone follow-up of postal non-respondents [15]. The aim of multiple postal reminders was to increase the response rate, whereas the telephone follow-up was designed to assess non-response bias and was not a part of the ordinary survey. The main outcome measures in this study were increase in response rate for each reminder, the effects of demographic and practice variables on response, the effects of non-response on survey estimates, and the cost-effectiveness of each reminder.

## Methods

### Sample

The study sample was randomly selected from a list of all GPs in Norway being a part of the Regular General Practitioner (RGP) scheme. The questionnaire was mailed to 1,400 regular GPs. Eight GPs were excluded from the survey because of incomplete practice address or other reasons for ineligibility (leave of absence, quitted as GP).

### Materials

The Norwegian Knowledge Centre for the Health Services conducted the postal survey. The first postal mailing was sent March 9, 2009. The letter included a recommendation to take part in the survey by the leader of the Norwegian Association of General Practitioners. Non-respondents were sent three postal reminders with 10-14 days between each. To assess non-response bias non-respondents were telephoned after four postal contacts, starting approximately three weeks after the last reminder. An external market research company (TNS Gallup) carried out the telephone follow-up using the Computer Assisted Telephone Interview (CATI) method.

Researchers at the Commonwealth Fund and Harris Interactive designed the four-page questionnaire, with advice and review by experts in each country [16,17]. It focused on indicators for primary care practice capacity to manage care well and on payment incentives to support quality improvement. The questionnaire consisted of the following core topics: health system views and practice satisfaction, access, patient care, teams, coordination of care, office systems and information technology, measuring practice performance, and financial support or incentives. The final English questionnaire was translated independently to Norwegian by two Norwegian researchers. The researchers reached an agreed upon version after review and discussions of the two separate translations. The final Norwegian translation was sent to three GPs in Norway to assess face validity. The questions have varying response formats, from a simple "yes or no" format to a Likert format with five response options.

The postal questionnaire was quite extensive consisting of 58 main questions in addition to ten questions about practice profile and demographical data. This combined with the fact that the telephone follow-up aimed to achieve responses from a difficult target group made us decide to use a short version of the postal questionnaire in the telephone interviews. The criteria for selection of questions for telephone interviews were: coverage of questionnaire domains; relevance in Norway; data quality as assessed by preliminary review of completed postal questionnaires. The selection process reduced the questionnaire from 58 to 23 questions. Because the telephone interviews are an important part of this study we chose to confine the study to the short version of the questionnaire (23 questions).

Demographic and practice related data about GPs came from the Norwegian Medical Association (NMA) and the Norwegian Labour and Welfare organisation; gender, age, number of years as GP, type of practice (group/solo), being a specialist in general practice medicine or not, available positions on GP list (yes/no), and practice address. Two independent variables were based on practice address; health region (South-East, West, Middle, North) and municipality size (< 5 000, 5-15 000, 15-50 000, > 50 000).

Costs relating to data collection were registered using an electronic internal accounting system. Costs related to the postal administration included printing, mailing and salaries for administrative staff organising mailings, receiving and scanning questionnaires. Since the telephone follow-up was conducted by an external market research company, the payment to them forms the cost of conducting the follow-up.

### Analysis

GPs were placed in one of five groups: group 1, respondents before the first reminder; group 2, respondents to the first reminder; group 3, respondents to the second reminder; group 4, respondents to the third reminder; and group 5, respondents to the telephone follow-up. It takes a minimum of five working days from sending a postal reminder to a GP and receipt of a completed questionnaire which was used to define the response group.

The cumulative response rate was assessed through the five survey phases. Response probability was assessed by a multiple logistic regression with response as dependent variable (yes/no) and eight demographic and practice related variables as predictors.

The effects of non-response bias on survey results were firstly assessed through linear regression. The 23 questions were grouped into six questionnaire topics: overall view of the health system and practice satisfaction; access; use of guidelines; coordination of care; office systems and information technology; measuring practice performance. The aim was to select one item from each topic, the main criteria being pure statistical; the variables should be suited as dependent variables in linear regression. Five of six topics included questions with three to five response options, and were selected as dependent variables in multiple linear regressions with eight demographic and practice variables as predictors. The five questions were recoded so that higher values represent a more positive evaluation. The second approach to analysing the effects of non-response bias on survey results consisted of a comparison of questionnaire scores for the five response groups using one-way analysis of variance (ANOVA).

Total costs were calculated for each postal respondent group. The cost-effectiveness of each reminder or follow-up was calculated by dividing total costs for each respondent group with total responses in each group.

SPSS version 15.0 was used for statistical analyses, except for cost calculations which used Microsoft Office Excel 2003.

## Results

### Response rate

Completed questionnaires were returned by 406 (29.2%) GPs before any reminders were received. The response rates increased to 42.7% (595), 50.4% (701) and 55.4% (771) after the first, second and third postal reminders, respectively. A further 3.7% (n = 52) completed a telephone-administered short version of the questionnaire, giving a total response rate of 59.1% (823) after five contacts.

The logistic regression showed that three of eight variables were significantly related to the response variable (table 1). GPs in group practice had higher probability of answering than GPs in solo practice (OR = 1.6, p = 0.01). The regression also showed that specialists in general practice medicine answered more often than non-specialists (OR = 2.0, p < 0.001), while GPs in the most rural municipalities answered more often than GPs in the most urban municipalities (OR = 1.6, p = 0.03).

**Table 1:** Logistic regression model: association between background variables and response

Variable	Odds ratio (OR)	OR 95% confidence interval	p
*Male (vs. female)*	1.02	0.78-1.33	0.90
*Mean age*	0.99	0.97-1.01	0.33
*Mean years as GP*	0.98	0.96-1.00	0.07
*Group practice (vs solo practice)*	1.62	1.12-2.33	0.01*
*Specialist (vs not specialist)*	2.04	1.51-2.76	<0.001***
*Available positions on GP list (vs not available)*	0.78	0.60-1.02	0.07
			
*Health region*			
West (vs South/East)	0.78	0.56-1.10	0.15
Middle (vs South/East)	1.14	0.77-1.70	0.51
North (vs South/East)	0.69	0.44-1.10	0.09
			
*Municipality centrality*			
Least central (vs most central)	1.61	1.10-2.44	0.03*
Little central (vs most central)	1.31	0.80-2.14	0.29
Quite central (vs most central)	1.02	0.75-1.40	0.91

Significance levels: ***p < .001,; **p < .01,; *p < .05

### Non-response and survey estimates

Linear regression analysis showed that the independent variables had a relatively weak association with the five dependent variables (table 2). In all five models the independent variables only explained a small amount of the variance in the dependent variables; from 3.5% for job satisfaction to 5.8% for discharge letter time and electronic laboratory results. Only two variables had more than one significant regression coefficients after controlling for the other independent variables; age was negatively associated with job satisfaction (B = -0.01, p < 0.001) and positively associated with perception of discharge letter time (B = 0.01, p < 0.001), while being a specialist in general practice medicine was negatively associated with job satisfaction (B = -0.15, p = 0.02), and positively associated with assessment of waiting time for specialist (B = 0.14, p = 0.03) and performance assessment (B = 0.18, p = 0.001).

**Table 2:** Linear regression models: association between demographic and practice variables and the five dependent variables

Variable	Job satisfaction		Waiting time for specialist		Discharge letter time		Electronic access to laboratory results		Receive information about performance compared to others	
	Coefficient	P^a^	Coefficient	P^a^	Coefficient	P^a^	Coefficient	P^a^	Coefficient	P^a^
Female (vs. male)	0.03	0.55	-0.13	0.01*	-0.04	0.44	-0.01	0.75	-0.00	0.97
Age	-0.01	0.001 **	-0.00	0.99	0.01	<0.001 ***	-0.00	0.73	-0.00	0.57
Years as GP	0.01	0.15	0.00	0.69	-0.00	0.72	0.00	0.84	0.00	0.31
Group practice (vs. solo practice)	0.04	0.63	-0.08	0.30	-0.08	0.30	0.24	<0.001 ***	-0.05	0.50
Specialist (vs. not specialist)	-0.15	0.02*	0.14	0.03*	0.01	0.89	0.07	0.06	0.18	0.001 **
Available positions on GP list (vs. not having positions available)	-0.08	0.14	-0.04	0.43	0.00	0.98	-0.03	0.30	0.01	0.90
										
Health region										
West (vs. South-East)	-0.12	0.07	-0.15	0.02*	-0.07	0.24	0.04	0.33	0.07	0.23
Middle (vs. South-East)	-0.05	0.50	-0.00	0.98	-0.05	0.48	0.02	0.67	-0.05	0.40
North (vs. South-East)	0.01	0.89	-0.20	0.02*	-0.09	0.29	0.07	0.18	-0.03	0.67
										
Municipality size										
< 5 000 (vs. > 50 000)	-0.09	0.27	-0.06	0.46	-0.04	0.54	0.06	0.18	-0.03	0.64
5-15 000 (vs. > 50 000)	-0.02	0.89	-0.14	0.14	-0.03	0.73	-0.09	0.11	-0.02	0.80
15-50 000 (vs. > 50 000)	-0.01	0.84	-0.11	0.06	-0.09	0.11	-0.01	0.76	0.02	0.70

Note: All models are significant (p < .05). R square range from 0.035 (job satisfaction) to 0.058 (discharge letter time and electronic laboratory results). In the regressions the dependent variables are recoded; higher values represent better responses.

^a ^***p < .001 **p < .01 *p < .05

The differences in item scores for the five groups of respondents were small (table 3). The largest difference between any of the groups on the five questions was only 0.2, this relating to the questions about job satisfaction, waiting time for specialist and performance measurement. One-way ANOVA tests showed that the only question with significant variation between the groups was waiting time for specialists (p = 0.03).

**Table 3:** Item scores for the five respondent groups

		Groups 1-4 postal respondents				
			
**Item**^a^	Total respondents (n = 823)	1 Initial respondents (n = 406)	2 First reminder (n = 189)	3 Second reminder (n = 106)	4 Third reminder (n = 70)	5 Telephone respondents **(n = 52)**^c^
Job satisfaction	3.3	3.3	3.2	3.2	3.2	3.4
Waiting time for specialist	1.5*	1.6	1.4	1.4	1.6	1.4
Discharge letter time	3.7	3.7	3.7	3.7	3.7	3.8
Electronic access to laboratory results	2.9	2.9	2.9	2.9	2.9	3.0
Receive information about performance compared to others	1.3	1.3	1.3	1.3	1.3	1.5

Note. One-way ANOVA tests. ***p < .001 **p < .01 *p < .05

a Items are recoded; higher values represent better responses.

### Survey costs

The total cost of data collection was €30 385 (table 4). The cost per response for each of the five groups was estimated to be €23.8 for group 1 (no reminders), €35.5 for group 2 (one reminder), €48.2 for group 3 (two reminders), €62.3 for group 4 (three reminders) and €87.4 for group 5 (telephone). The percentage of the total survey costs was 31.8%, 22.1%, 16.8%, 14.4% and 15% for the five groups respectively.

**Table 4:** Costs for the five respondent groups

		Groups 1-4 postal respondents				
			
Variable	Total respondents (n = 823)	1 Initial respondents (n = 406)	2 First reminder (n = 189)	3 Second reminder (n = 106)	4 Third reminder (n = 70)	5 Telephone respondents (n = 52)
Total costs for each respondent group (€)	-	9 655	6 718	5 108	4 362	4 543
Cumulative survey costs (€)	-	9 655	16 372	21 479	25 841	30 385
Percentage of survey costs in each respondent group	-	31.8	22.1	16.8	14.4	15.0
Costs per response for each respondent group (€)	-	23.8	35.5	48.2	62.3	87.4

## Discussion

Compared to other relevant studies the Norwegian survey reached an acceptable response rate. The final response rate was five percent higher than the average response rate in physician surveys in the study by Asch et al. [2], seven percent higher than the average response rate for large physician surveys with over 1,000 observations in the study by Cummings et al. [3], and higher than nine of ten other countries in the Commonwealth Fund survey in 2009 [17].

However, the central issue in surveys is not response rate in itself but the degree of non-response bias [2,3]. Very few studies have assessed non-response bias. In the study by Cumming et al. [3], only 18% of the articles performed any type of comparison between respondents and non-respondents. In another study of 350 studies from 1996 to 2005, Cook et al. found that only 17% reported some form of non-response analysis [10]. The current study assessed the effect of non-response using available methods. First, there was a range of variables included within the sampling frame which were used to assess variables related to non-response. Only three variables had a significant association with the response variable, and these variables were not at all or only weakly related to the main dependent variables. Secondly, results for postal respondents and telephone respondents were compared, the latter group giving estimates for postal non-respondents. Small and largely non-significant differences were found between the five respondent groups. Therefore, both methods for analysing non-response bias indicate a small bias. Of course, it cannot be known with certainty whether non-response has introduced bias, but available methods were used to assess the likelihood of bias. Together with an acceptable response rate and the available sampling frame for statistical weighting, there are sufficient reasons to conclude that the survey results can be generalized to the population of GPs in this survey.

The Commonwealth Fund health policy survey was conducted in eleven countries in 2009 [17]. The findings in the current study indicate adequate representativeness in the Norwegian sample, but the validity of this finding for the ten other countries needs to be considered. Both the data collection methods and the response rate varied considerably between the eleven countries; data was collected by both postal survey, telephone interview and email survey, and the number of reminders, incentive structure and several other factors varied [17,18]. Therefore, it is difficult to generalize the Norwegian findings about non-response bias to all countries. However, they should be most relevant for countries using approximately the same data collection methods. This primarily relates to The Netherlands and Sweden who used a postal survey with multiple reminders, but also Germany, Australia, New Zealand, Canada and USA used this approach but also offered an incentive to the invited primary care physicians. The response rate is far below 50% for USA and Canada, a marked difference from the other countries using a postal design. However, the findings in the present study show that estimates from the first 30% of GPs are close to the item scores for all respondents. This is in accordance with other relevant studies [15,19-21]. Consequently, the low response rates in USA and Canada have the potential of constituting a representative survey, but organizational or cultural differences needs to be further considered. The validity of comparing Norwegian results from the current study with data from France, Italy and United Kingdom who used a different data collection mode, is more uncertain, indicating the need for further research.

The postal questionnaire consisted of 58 main questions. The telephone interviews were reduced to 23 questions because non-respondents after four postal contacts were considered a difficult target group. The criteria for selection of questions for telephone interviews were coverage of questionnaire domains, relevance in Norway and data quality as assessed by preliminary review of completed postal questionnaires. The 23 questions were grouped into six questionnaire topics in this study, and then we selected five questions from five topics based on statistical criteria. A relevant question concerns the validity of findings for the questions not included in this study. Results not shown here confirm that the analysis in table 2 and 3 with the other questions from the telephone interviews in large degree coincide with the findings in this study, indicating validity for all questions in the questionnaire. The only exception is related to questions about the GPs' use of clinical guidelines; telephone respondents had systematically higher scores than other respondent groups. This finding coincides with other studies and indicates a social desirability bias in telephone interviews [13-15], whereby respondents over-report positive behaviour. This finding implies that the value of assessing non-response bias by means of telephone follow-ups of postal non-respondents is restricted for questions affected by social desirability. It also raises concerns about the validity of comparing countries using different data collection methods, especially postal vs. telephone modes and especially for questions affected by social desirability. This issue warrant further research, but in general we recommend standardized data collection methods in international surveys.

The cost-effectiveness of the final reminders was poor and the survey cost would have been almost halved without the last three reminders. Ending data collection after the first postal reminder would have produced the same results for the main variables, at the same time 72% of the respondents would have been secured. Other studies have also found small differences in results between early and late respondents [15,19-21]. Therefore, after considering a broader set of criteria it might be argued that a more modest approach to postal data collection in GP surveys could be a fruitful approach, especially in studies where costs and time are limiting factors. This would also be more sensitive to the high practice workload of GPs which constrains their participation in surveys [12].

## Conclusions

The response rate in the Norwegian survey was satisfactory, and the effect of non-response was small indicating adequate representativeness. The cost-effectiveness of the final reminders was poor. The Norwegian findings strengthen the international project, but restrictions in generalizability warrant further study in other countries.

## Competing interests

The authors declare that they have no competing interests.

## Authors' contributions

OAB planned the paper together with HII and GBU, carried out the statistical analysis, conducted the study together with administrative staff, and drafted the paper. HII planned the study together with OAB and GBU, and revised the draft critically. GBU planned the study together with OAB and HII, and revised the draft critically. All authors read and approved the final manuscript.

## Pre-publication history

The pre-publication history for this paper can be accessed here:

http://www.biomedcentral.com/1472-6963/10/38/prepub
